# Neurofibromatosis Type Two: A Case With Both Intracranial and Spinal Lesions

**DOI:** 10.7759/cureus.20535

**Published:** 2021-12-20

**Authors:** Sandipta Banerjee, Ajay Agarwal

**Affiliations:** 1 Department of Neurosurgery, Kolkata Medical College & Hospital, Kolkata, IND; 2 Department of Neurosurgery, The Calcutta Medical Research Institute, Kolkata, IND

**Keywords:** neurofibromatosis type two, retromastoid craniectomy, tumor excision with laminectomy, spinal tumors, vestibular schwannoma

## Abstract

We discuss the case of a 36-year-old male patient who presented with gait impairment. On examination, he had clinical findings of cervical myelopathy. The patient was evaluated with an MRI of the brain and spine, which revealed multiple spinal tumors, some causing significant canal stenosis. The spinal tumors involved the cervical, thoracic, and lumbar regions. There were both intramedullary and extramedullary tumors with an extraspinal extension. The patient's MRI brain also revealed bilateral vestibular schwannomas. His family history was negative. He subsequently underwent surgery for multiple spinal lesions followed by debulking of the right-sided vestibular schwannoma. The radiological findings of both intramedullary and extramedullary spinal tumors affecting the spinal cord and extensively involving the cervical, thoracic, and lumbar regions, and the requirement of spinal and cranial surgery concurrently make this a challenging neurosurgical case.

## Introduction

Neurofibromatosis is a genetic disorder of the nervous system that causes tumors to form in the brain, spinal cord, and nerves [1]. The condition is further classified into neurofibromatosis type I (NF1) and neurofibromatosis type II (NF2). NF1 presents with a variety of characteristic abnormalities of the skin and the peripheral nervous system, while NF2 presents with vestibular schwannomas, meningiomas, and gliomas [2]. The diagnosis of NF2 is usually made in the second and third decades of life, and most patients are aged 18-24 years. The patients with a family history of NF2 should be screened as early as 10-12 years of age followed by annual MRI screening until they reach 40 years of age [1]. NF2 is caused by a mutation on chromosome 22 [3]. The NF2 gene product is known as Merlin and it is a tumor suppressor. Its decrease/absence causes a predisposition to the development of various tumors in the central and peripheral nervous systems. Tumors are generally benign but may produce debilitating myeloma depending on their location. Diagnosis is essentially based on clinical findings and imaging. Biopsy, if performed, is supportive, and genetic testing is rarely necessary. There is no prevention or cure for this condition. Surgery remains the focus of current management although watchful waiting and, in some cases, radiation treatment has a role. Currently, we are witnessing the advent of tailored drug therapies aimed at the genetic level and these are likely to provide huge improvements in the treatment of this devastating, life-threatening condition [4].

## Case presentation

**Figure 1:**
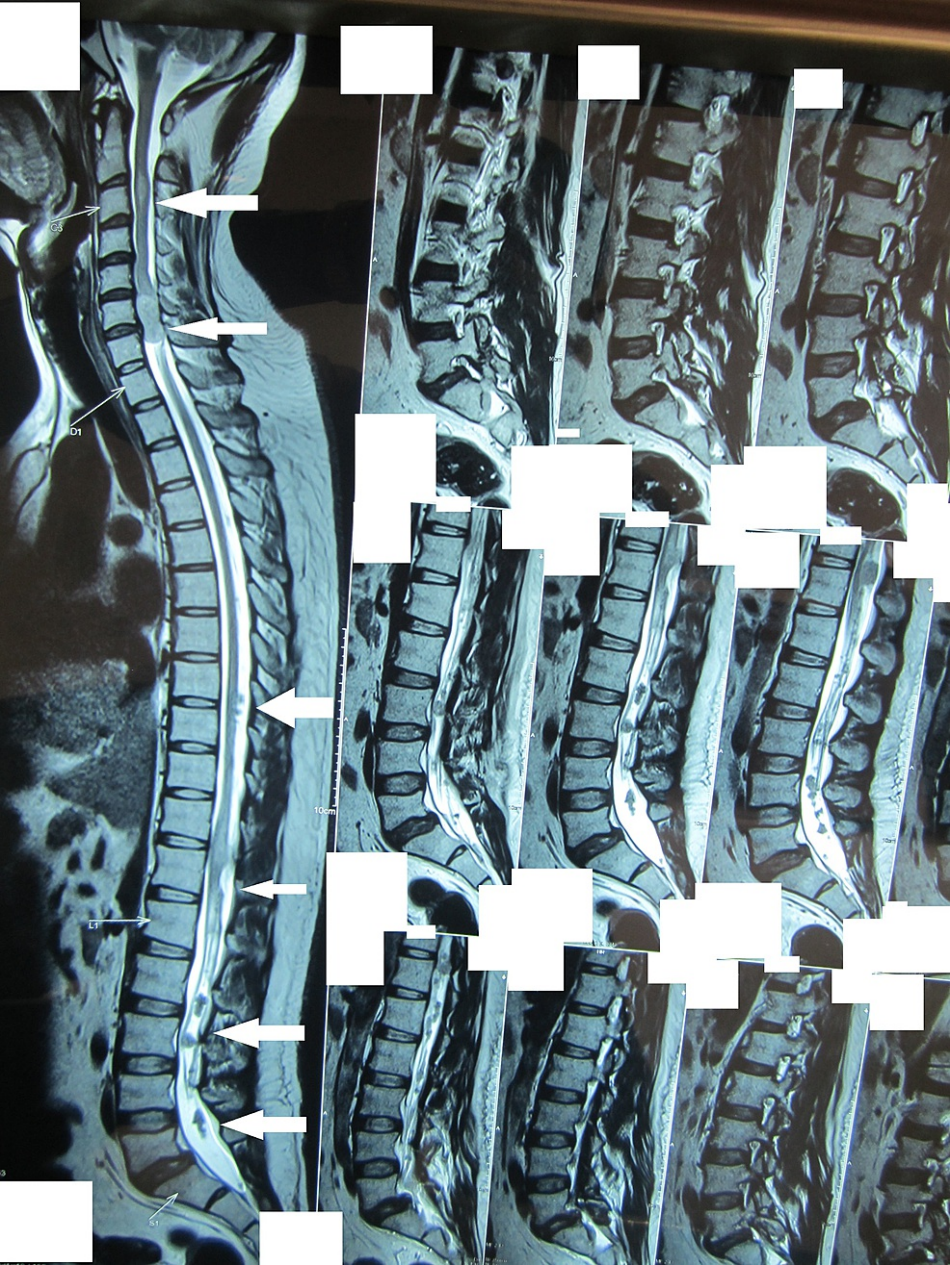
MRI sagittal section T2 sequence showing multiple tumors in different regions of the spinal cord (arrows) MRI: magnetic resonance imaging

**Figure 2:**
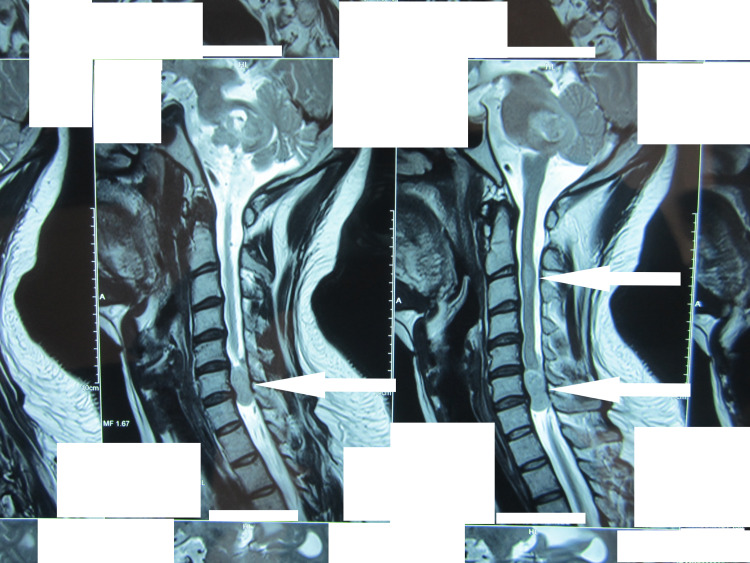
MRI showing multiple tumors in the cervical region of the spinal cord (arrows) MRI: magnetic resonance imaging

**Figure 3:**
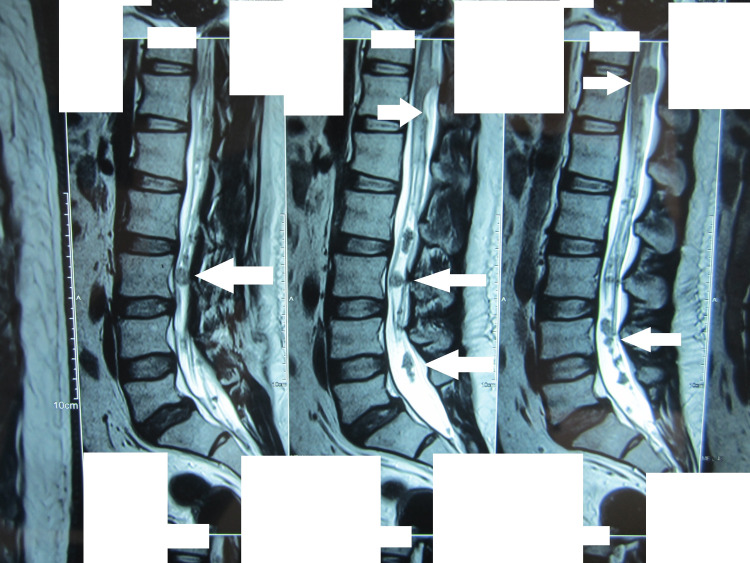
MRI of the lumbosacral region without contrast in T2 sequence showing multiple tumors compressing the spine in the lower thoracic and lumbar regions (arrows) MRI: magnetic resonance imaging

**Figure 4:**
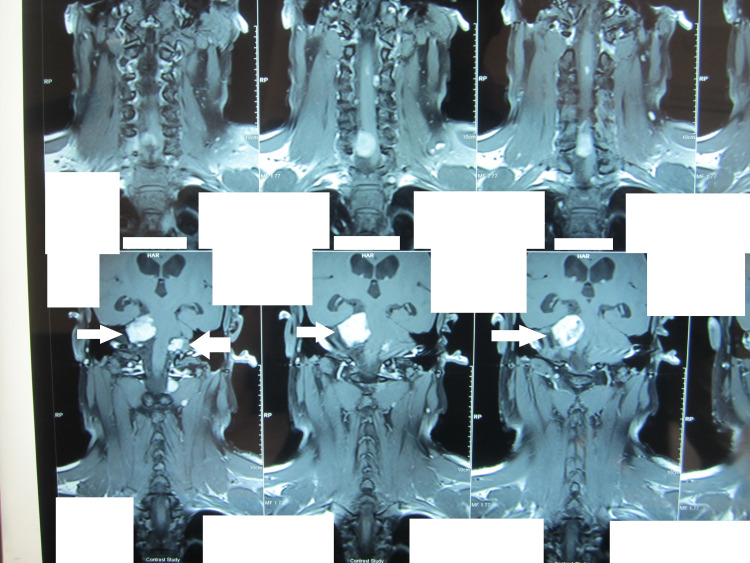
MRI brain/cervical spine coronal section without contrast in T2 sequence showing bilateral vestibular schwannomas (arrows) MRI: magnetic resonance imaging

A 36-year-old male patient presented with progressive weakness of the lower limbs for two months. Additionally, tingling, numbness, and stiffness were present. He had previously consulted his local doctor when he had reached a stage where he had been unable to walk, and he had been advised to undergo an MRI of the spine. The patient was a known diabetic and hypertensive. The MRI of the spine had revealed multiple spinal tumors (Figures 1, 2, 3). The MRI had also incidentally found bilateral cerebellopontine (CP) angle tumors (Figure 4). He had then been referred for neurosurgical advice.

On further questioning, he stated that he did have a degree of hearing loss on the right side, which he had noticed for the past six months, and a mild headache, which was more recent in origin. He did not have any family history suggestive of NF2. On examination, he was oriented to person, place, and time. His speech was also normal. Cranial nerve examination revealed that the functions of the cranial nerves 1-6 were not affected. However, there was cranial nerve 7 (CN VII) involvement in the form of right facial asymmetry [lower motor neuron (LMN) type, grade 1] and loss of taste sensation on the anterior two-thirds of the tongue. For cranial nerve 8 (CN VIII) examination, the Rinne test was performed. Air conduction was greater than bone conduction on both sides and the Weber test lateralized to the left ear. This signified sensorineural hearing loss on both sides with greater severity on the right side. The functions of the other cranial nerves were within normal limits. Regarding motor functions, the nutritional status of all four limbs was normal. Both the upper limbs had a normal tone. Clasp-knife spasticity was present in both the lower limbs. The power of the proximal and distal left upper limbs was normal (grade 5). The power of the proximal right upper limb was grade 5 while that of the distal right upper limb was grade 3. For the lower limbs, the power was grade 2 for the proximal and distal parts of both sides. Cerebellar signs - dysmetria and dysdiadochokinesia - were present on the right side. As for deep tendon reflexes (DTRs), the biceps jerk on both sides was within normal limits. On the right side, triceps jerk and brachioradialis jerks were lost. However, on the left side, they were within normal limits. DTRs in the lower limb, such as the knee and the ankle jerks, were brisk. Plantar was upgoing on both sides. Also, the patient did not have any sensory loss along the affected dermatomes. However, posterior column sensations including vibration and proprioception were affected in the lower limb. The gait could not be tested due to the low power of muscles in the lower limb.

The patient's MRI spine without contrast revealed multiple spinal tumors in the cervical, thoracic, and lumbar areas (Figures 1-3). Of note were two tumors, one at C6-7 levels, which was extending into the right neural canal and measuring 3.6 x 2.4 x 8.5 cm in size, and another one at T11-12 levels; both were extramedullary extradural but causing significant cord compression due to their size. There was an intramedullary tumor in the cervical region (Figure 1). MRI brain showed a sizeable mass in the right CP angle, which was extending into the internal acoustic canal and causing cerebellar and brain stem compression. A small mass extending into the left acoustic foramen was also seen, which did not cause any compression on the adjacent structures (Figure 4).

Excision of the cervical and dorsal tumors was done in the same setting via a C6-7 and T11-12 laminectomy respectively. Postoperatively, the patient had significant improvement in his lower limb power (grade 4) and gait.

**Figure 5:**
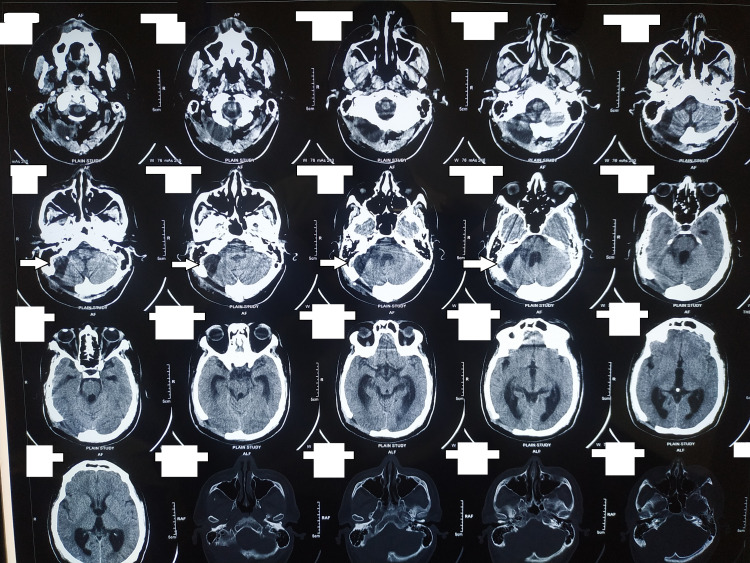
Postoperative CT scan of the brain CT: computed tomography

Biopsy of both the tumors suggested schwannoma. Almost a month later, the patient underwent a right retromastoid craniectomy with debulking of the right vestibular schwannoma. A near-total excision was performed, leaving the residual tumor around the internal meatus with anatomical preservation of CN VII (Figure 5). Postoperatively, the patient had improvement in his dysmetria and dysdiadochokinesia but had aggravation of the right CN VII paresis. Histopathology was consistent with schwannoma. He was advised to undergo Gamma Knife radiosurgery for the residual on the right and the tumor on the left side.

## Discussion

NF2 is inherited as an autosomal-dominant condition [1]. Of note, 50% of the cases are autosomal-dominant, while the remaining 50% are associated with a de-novo mutation. Our patient did not have any relevant family history. Tumors other than vestibular schwannomas are also known to be associated with a poor prognosis in patients with NF2 [5]. Surgery is the main method of treatment in the case of spinal tumors. Tumor resection should be planned when radiological tumor progression is evident, even if the spinal tumors are asymptomatic, to preserve neurological function and ensure good quality of life.

At the time of his presentation, our patient was symptomatic for both cranial and spinal lesions. Although the presentation of this patient was symptomatic and slowly progressive, patients can be asymptomatic on presentation in some cases [6]. Patients with both bilateral vestibular schwannomas and spinal lesions generally present with cranial symptoms. However, there is scarce literature on the temporal relation of the presentation of cranial and spinal symptoms among patients. The time gap between the cranial and spinal surgery, in patients undergoing both, also could not be ascertained. Our patient had a fairly large right-sided vestibular schwannoma (6 x 11 x 9 cm) that was compressing the brainstem (Figure 4). Simultaneously, the cervical spine tumor had almost obliterated the entire canal (Figure 2). Both surgeries that we performed were necessary for the relief of symptoms.

Spinal tumors occur in 89% of cases of NF2. However, multiplicity is found in about 56% of these [5]. There is no predilection for any region of the spine, and the cervical, thoracic, or lumbar region could be affected. Extramedullary lesions can be meningiomas, neuromas, and schwannomas, and they constitute the majority of spinal lesions. Intramedullary lesions, on the other hand, constitute less than one-third of the spinal lesions. Most of the lesions are small or asymptomatic. Symptomatic spinal lesions that require surgery have been reported in various studies at a rate of 30% or less. Our patient had both intramedullary and extramedullary lesions, including a dumbbell lesion in the cervical spine. Tumors were present in the cervical, thoracic, and lumbar regions.

## Conclusions

We described the case of a patient who presented with concurrent tumors in the brain (bilateral vestibular schwannomas) and the spinal cord. The spinal tumors were found in the cervical, thoracic, and lumbar regions. It is unique to have such a large area of involvement. Also, both intramedullary and extramedullary (both extradural and intradural) spinal tumors were noted in this patient. The requirement of spinal and cranial surgery almost concurrently makes this case challenging and interesting.
